# How to identify sex chromosomes and their turnover

**DOI:** 10.1111/mec.15245

**Published:** 2019-10-10

**Authors:** Daniela H. Palmer, Thea F. Rogers, Rebecca Dean, Alison E. Wright

**Affiliations:** ^1^ Department of Animal and Plant Sciences University of Sheffield Sheffield UK; ^2^ Department of Genetics, Evolution and Environment University College London London UK

**Keywords:** bioinformatics, next‐generation sequencing, sex chromosome turnover, sex chromosomes

## Abstract

Although sex is a fundamental component of eukaryotic reproduction, the genetic systems that control sex determination are highly variable. In many organisms the presence of sex chromosomes is associated with female or male development. Although certain groups possess stable and conserved sex chromosomes, others exhibit rapid sex chromosome evolution, including transitions between male and female heterogamety, and turnover in the chromosome pair recruited to determine sex. These turnover events have important consequences for multiple facets of evolution, as sex chromosomes are predicted to play a central role in adaptation, sexual dimorphism, and speciation. However, our understanding of the processes driving the formation and turnover of sex chromosome systems is limited, in part because we lack a complete understanding of interspecific variation in the mechanisms by which sex is determined. New bioinformatic methods are making it possible to identify and characterize sex chromosomes in a diverse array of non‐model species, rapidly filling in the numerous gaps in our knowledge of sex chromosome systems across the tree of life. In turn, this growing data set is facilitating and fueling efforts to address many of the unanswered questions in sex chromosome evolution. Here, we synthesize the available bioinformatic approaches to produce a guide for characterizing sex chromosome system and identity simultaneously across clades of organisms. Furthermore, we survey our current understanding of the processes driving sex chromosome turnover, and highlight important avenues for future research.

**Glossary**
Achiasmy: Complete recombination suppression in one sex.
Coverage: Number of DNA‐seq reads that represent a given nucleotide in a reference genome. For autosomal regions, coverage can be calculated as *N* × *L*/*G*, where *N* is the number of reads, *L* is read length, and *G* is the length of the reference genome.
Dosage compensation: A mechanism to maintain ancestral expression levels of the X or Z chromosome relative to the autosomes in the heterogametic sex. This is thought to evolve in response to degeneration of the sex‐limited chromosome and subsequent unequal gene dose between males and females.
Heteromorphic sex chromosome: Sex chromosomes that are karyotypically highly distinct from each other. The X and Y (or Z and W) chromosomes are diverged and show differences in gene content and size.
Homomorphic sex chromosome: Sex chromosomes that are nearly identical in gene content and size. They are more challenging to identify from cytogenetic data alone.
*k*‐mer: All possible subsequences of a given length *k* within a genome.
Pseudoautosomal region (PAR): Homologous region of the sex chromosomes that continues to recombine between the X and Y (or Z and W).
Restriction site‐associated DNA (RAD) sequencing: A restriction site‐associated DNA sequencing technique. A restriction enzyme is used to digest genomic DNA into fragments which are then ligated to adapters that will bind to an Illumina flow cell. Both ends of these fragments are then sequenced using next‐generation methods.
Stratum: Region where recombination between the sex chromosomes has been halted.
Synteny: Conserved collinear regions. Conservation of gene order across two sets of chromosomes that are being compared to each other.

## 1 INTRODUCTION

Sexual reproduction is a fundamental feature of eukaryotes, yet the mechanisms by which sex is determined are highly diverse (Bachtrog et al., 2014; Beukeboom & Perrin, 2014; Bull, 1983). This variation is apparent even among closely related species, or populations of the same species (Tree of Sex Consortium, 2014). In many organisms, sex chromosomes are associated with male or female development, and in many groups, including birds (Zhou et al., 2014), eutherian mammals (Cortez et al., 2014) and certain insects (Fraïsse, Picard, & Vicoso, 2017), the sex chromosome system is stable and highly conserved. However, it is apparent that sex chromosomes often evolve rapidly in many lineages, and the chromosome pair that determines sex can change rapidly over time (Pennell, Mank, & Peichel, 2018). In addition to turnover in the chromosome pair recruited to determine sex, transitions between different sex chromosome systems (e.g., XY to ZW, or ZW to XY) are also well documented across numerous clades. This diversity is particularly pronounced in certain groups of reptiles (Gamble et al., 2015; Pokorná & Kratochvíl, 2009), amphibians (Jeffries et al., 2018), fish (Darolti et al., 2019; Kitano & Peichel, 2012; Mank, Promislow, & Avise, 2006), insects (Blackmon & Demuth, 2014; Vicoso & Bachtrog, 2015) and plants (Balounova et al., 2019; Martin et al., 2019; Tennessen et al., 2018), where turnover between male (XY) and female (ZW) heterogamety is common over relatively short evolutionary time periods (Pennell et al., 2018). While recent efforts, including those of the Tree of Sex Consortium, have focused on characterizing the tremendous diversity of sex chromosomes across species, it is clear that we currently have an incomplete understanding of the variation in sex determination mechanisms across the tree of life (Bachtrog et al., 2014; Tree of Sex Consortium, 2014).

Despite the growing awareness that sex chromosomes have evolved independently many times throughout eukaryotes, our understanding of the processes driving the formation and turnover of new sex chromosome systems is limited and many unanswered questions remain. A large body of theoretical work outlines predictions for when and why sex chromosome transitions occur (Beukeboom & Perrin, 2014), including genetic drift (Bull & Charnov, 1977; Saunders, Neuenschwander, & Perrin, 2018), mutation load on the sex‐limited chromosomes (Blaser, Grossen, Neuenschwander, & Perrin, 2013; Blaser, Neuenschwander, & Perrin, 2014), selection on sex ratio (Jaenike, 2001; Werren & Beukeboom, 1998) and sexually antagonistic selection (van Doorn & Kirkpatrick, 2007, 2010), yet attempts to empirically test these have been restricted to a few clades (Blackmon & Demuth, 2014; Jeffries et al., 2018; Kitano & Peichel, 2012; Wright et al., 2017). Identifying the evolutionary and genomic mechanisms predicted to drive sex chromosome turnover is a major priority, which in turn will shed light on why sex determination is labile in some taxa and not in others. Furthermore, differences in transmission pattern between male and female heterogametic sex chromosome systems (Beukeboom & Perrin, 2014) are predicted to have important consequences for adaptation (Mank, Vicoso, Berlin, & Charlesworth, 2010; Wright et al., 2015), sexual dimorphism (van Doorn & Kirkpatrick, 2010; Mullon, Wright, Reuter, Pomiankowski, & Mank, 2015; Muralidhar, 2019), and ultimately speciation (Irwin, 2018; Mank et al., 2010). Efforts to rigorously test predictions about the causes and consequences of sex chromosome evolution have been largely hampered by our incomplete knowledge of the diversity of sex chromosomes across a broad taxonomic range and limited power to identify convergent trends across independently evolved sex chromosomes. Traditionally, cytogenetic methods have been used to identify sex chromosome systems and turnover events (Valenzuela, Adams, & Janzen, 2003). However, while there have been recent improvements that facilitate sex chromosome identification using these approaches (Ezaz et al., 2005; Iannucci et al., 2019; Kawai et al., 2007), identifying homomorphic sex chromosomes, where the pair are nearly identical in gene content and size, is still challenging. This might disproportionately affect the identification of ZW systems as W chromosomes are predicted to evolve more slowly than Y chromosomes (Bachtrog et al., 2011), resulting in the underestimation of turnover events. To address how, when, and why sex chromosomes evolve (Wright, Dean, Zimmer, & Mank, 2016) we require far more information on sex chromosomes in diverse clades.

**Figure 1:**
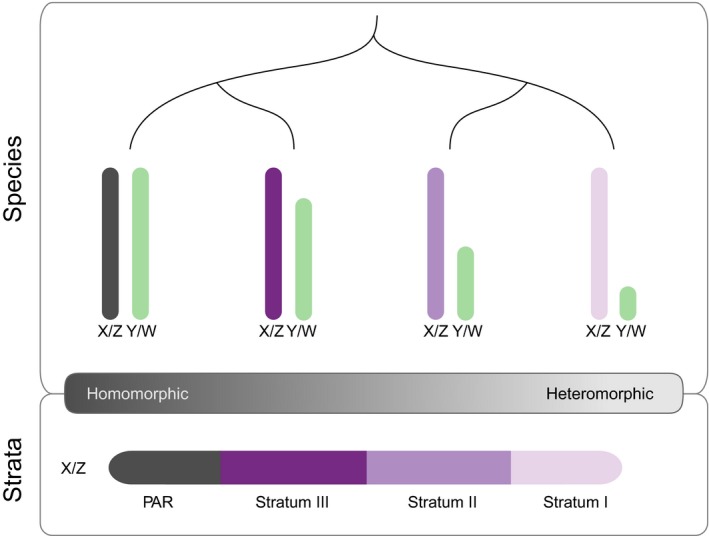
Illustration of the homomorphic‐heteromorphic sex chromosome continuum. Sex chromosomes can range from heteromorphic, where the X and Y (or Z and W) chromosomes are diverged and highly distinct, to homomorphic, where pairs are nearly identical in gene content and size. However, sex chromosomes can vary in their degree of sequence differentiation not just among species (top panel) but also among strata within a species (bottom panel). Strata are regions of the chromosome where recombination between the sex chromosomes has been halted independently and therefore are of different ages. Different methods for identifying sex‐linked loci will be appropriate for species/strata at different points on this continuum. Purple scale indicates sequence differentiation between chromosomes or strata, where lighter purple shows greater divergence

Recently, new bioinformatic methods are making it possible to identify and characterize sex chromosomes in a diverse array of non‐model species using next generation sequencing data. In combination with comparative phylogenetic analyses, it is now possible to rigorously test theoretical predictions for sex chromosome formation and turnover. However, despite the diversity of newly developed methods to identify sex chromosomes, there have been limited attempts to synthesize them into a comprehensive guide applicable to a wide range of organisms (but see Muyle, Shearn, & Marais, 2017). This is key because the effectiveness of different approaches is influenced by a number of factors. In particular, the degree of sequence divergence between the sex chromosomes is an important element to consider. Sex chromosomes evolve from a pair of identical autosomes as recombination between the X and Y (or Z and W) is suppressed (Charlesworth, Charlesworth, & Marais, 2005). Recombination cessation catalyzes sequence divergence between the sex chromosomes, which can ultimately lead to heterogametic chromosomes that show major differences in size and gene content with severely degenerated W or Y chromosomes (Charlesworth & Charlesworth, 2000). In contrast, homogametic sex chromosomes are almost identical and exhibit few differences from each other in gene content. It is important to note that homogamety and heterogamety are not discrete states and instead represent two extremes on a continuum of sex chromosome divergence (Figure 1). Certain bioinformatic approaches to identify sex chromosomes are more effective for species at different points on this continuum. In addition, while sex chromosomes across species exhibit variation in the degree of heterogamety, different regions of the same sex chromosome can also fall at different points along this continuum (Figure 1). This is because recombination is often suppressed in a stepwise process, resulting in strata of different ages (Charlesworth et al., 2005; Lahn & Page, 1999; Wright, Moghadam, & Mank, 2012). Therefore, a combination of different, complementary methods is often necessary to identify sex chromosomes, and sex‐linked regions, among species.

Here, we review the range of available approaches to identify sex chromosomes and fill in gaps across the tree of life, highlighting the strengths and weaknesses of each. We do not cover methods for high resolution sequencing of sex‐limited chromosomes, as these have been discussed elsewhere (Tomaszkiewicz, Medvedev, & Makova, 2017), but instead focus on producing a guide for characterizing sex chromosome system and identity across diverse clades. In turn, we discuss future priorities in sex chromosome research and suggest how to use this growing data set to test, highlighting the strengths and weaknesses of each, how and why sex chromosomes evolve.

## 2 GUIDE FOR IDENTIFYING SEX CHROMOSOMES

### 2.1 Genomic coverage approach

**Figure 2:**
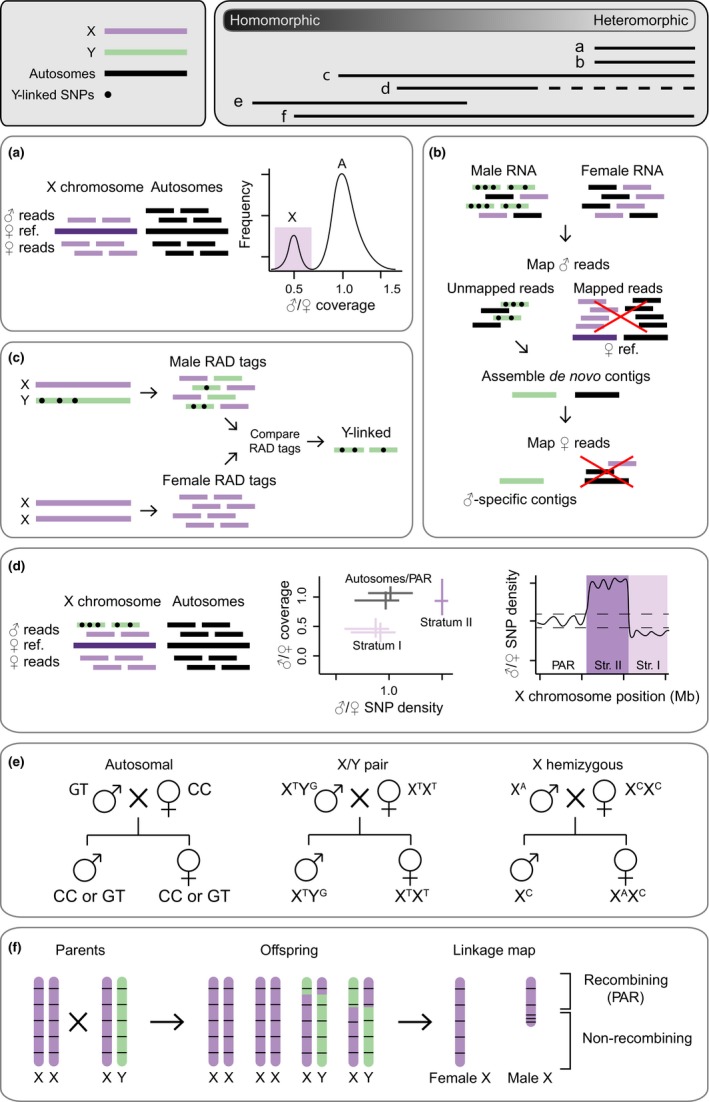
Overview of bioinformatic methods available for sex chromosome identification. This figure is based on XY sex chromosomes, but all methods can be inverted for ZW systems. Top left panel shows the key. Top right panel solid bars show which methods are most effective along different points of the sex chromosome divergence continuum. Dashed bar indicates that the method is partially effective. (a) Genomic coverage approach: in nonrecombining regions of sex chromosomes, where the Y has degenerated, males have only one X chromosome, and thus show a reduced genomic coverage relative to females. (b) Expression‐based approach: male RNA‐seq reads are mapped to a female reference. Unmapped reads are assembled into de novo contigs to identify putative Y‐linked sequences. Re‐mapping female transcripts to these contigs can be used to verify male‐limitation. (c) Association‐based approach: male and female RAD‐tags are compared to isolate male‐specific RAD loci. (d) SNP density approach: in younger regions of the sex chromosomes, which still retain high sequence similarity between the X and the Y, we expect an increase in male SNP density compared to females, as Y reads, carrying Y‐specific SNPs, still map to the homologous X regions. This SNP density pattern is not expected in old strata with substantial Y degeneration, as the X is effectively hemizygous in males. Contrasting sex differences in coverage and SNP density is a powerful approach to identify sex‐linked regions. (e) Segregation analysis approach: SNP data obtained from parents and progeny are analyzed in a statistical framework to assess the likelihood of autosomal versus sex‐linked segregation patterns. (f) Linkage mapping approach: recombination patterns of parents and offspring are compared, and regions with no recombination between males and females indicate putative sex‐linked regions

A common approach to identify sex chromosomes is based on genome coverage from next‐generation sequencing data. This approach exploits the difference in sex chromosome ploidy between males and females. In XY systems, X‐linked genes show half the number of genomic reads in males relative to females, and Y‐linked reads are absent in females (Figure 2a). This can be easily applied to ZW systems, where instead the W is absent in males, and females have only one copy of the Z. Since this approach is based on sex differences in genomic coverage, it is only effective when there is substantial sequence divergence between the sex chromosomes. Therefore, while it can be used to identify heteromorphic sex chromosomes or old, diverged strata, this method will misclassify pseudoautosomal regions, homomorphic sex chromosomes, or young strata as autosomal.

There are three main methods that employ genome coverage to distinguish sex chromosomes from autosomes. In the subtraction‐based method, DNA‐seq data from the homogametic sex are aligned to a reference genome generated from a heterogametic individual. As male and female genomes differ only by the Y (or W) chromosome, scaffolds with low coverage can be inferred as Y‐linked (or W‐linked). Whilst this approach can effectively identify sex‐limited scaffolds, and therefore establish whether the sex chromosome system is male or female heterogametic, it has limited potential for identifying the X or Z. This step is key for establishing the identity of the sex chromosome pair via synteny‐based approaches with other species (see Box 1), as sex‐limited chromosomes are often highly degenerated which hinders attempts to infer orthology. Alternatively, the ratio of male to female reads aligned to a reference genome can be used to directly distinguish X from autosomal scaffolds (Darolti et al., 2019; Vicoso & Bachtrog, 2011, 2013; Vicoso, Emerson, Zektser, Mahajan, & Bachtrog, 2013). For example, in an XY system, the male to female coverage ratio for autosomal and X scaffolds should be roughly 1 and 0.5 respectively. A variant of this method is called the chromosome quotient (CQ) approach (Hall et al., 2013). Due to noise in mapping reads to a genome, the male to female coverage ratio is typically a continuum, where there are two overlapping normal distributions of sex differences in coverage, one for the X or Z chromosome and the other for autosomal scaffolds (Figure 2a). Identifying the equidistant point between the maximum of these two peaks can help minimize the error in identifying sex‐linked regions, and has been employed successfully across a number of species (Huylmans, Toups, Macon, Gammerdinger, & Vicoso, 2019; Vicoso & Bachtrog, 2015). Lastly, the *k*‐mer counting approach (Akagi, Henry, Tao, & Comai, 2014; Carvalho & Clark, 2013; Li et al., 2018; Morris, Darolti, Bloch, Wright, & Mank, 2018; Pucholt, Wright, Conze, Mank, & Berlin, 2017) is based on similar underlying principles. Male and female genomes are broken up into *k*‐mers, counted computationally, and autosomal, Y‐, and X‐linked *k*‐mers are identified on the basis of read coverage. This method is unaffected by differences in filtering and read length and can be useful for identifying sex chromosomes across species where next‐generation sequencing data sets are of varying quality (Morris et al., 2018). Additionally, *k‐mer* analyses have been used to provide insight into the amount of repetitive elements accumulating on recently evolved Y chromosomes (Carvalho & Clark, 2013; Morris et al., 2018; Pucholt et al., 2017). Finally, in combination with next‐generation sequencing data obtained from flow‐sorted Y chromosomes, *k‐mer* approaches can filter contaminant autosomal and X‐linked sequences, thus improving the quality of the downstream Y chromosome assembly (Rangavittal et al., 2018).

However, there are a number of important caveats to consider. Coverage approaches are heavily sensitive to the algorithms used to map reads to a reference genome. This is because heteromorphic sex chromosomes still retain sequence orthology between the X and Y, and incorrectly mapped reads can mask coverage differences between the sexes and lead to the misclassification of sex‐linked sequences as autosomal. Stringent mapping parameters are recommended to minimize false negatives, with a maximum mismatch of 0 or 1 (Carvalho & Clark, 2013; Hall et al., 2013; Smeds et al., 2015; Vicoso et al., 2013), as well as the filtering of nonuniquely mapped reads (Vicoso & Bachtrog, 2015). Furthermore, repetitive regions of DNA should be masked prior to implementing these approaches to remove repeats shared by the sex‐limited chromosome and the autosomes (Carvalho & Clark, 2013; Hall et al., 2013; Smeds et al., 2015; Vicoso & Bachtrog, 2015). A similar caveat applies to the *k*‐mer approach, where *k*‐mer size can dramatically affect the number of inferred sex‐linked scaffolds. In principle, a large *k* ensures that identical *k*‐mers rarely result from sequencing errors and increases the probability that sequences encompass sex‐limited sites. However, if *k* is too large then *k‐mer* depth may be too low to detect statistical sex differences. In contrast, very short *k‐mers* are likely to be overrepresented in the data set, leading to low resolution to identify sex‐limited regions (Kelley, Schatz, & Salzberg, 2010). The choice of optimal *k*‐mer size can range from 15–31 bp depending on genome size of the organism (Carvalho & Clark, 2013; Morris et al., 2018). Coverage‐based approaches have been used to identify sex chromosomes from DNA‐seq data obtained from only one individual from each sex (Vicoso & Bachtrog, 2013) but read depth must be reasonably high to avoid confounding effects of sequencing errors (see Box 1) (>20‐fold; Carvalho & Clark, 2013; Hall et al., 2013; Smeds et al., 2015; Vicoso & Bachtrog, 2015). In practice, multiple individuals of each sex are required to avoid falsely identifying rare SNP variants as sex‐linked contigs, the probability of which will depend on the genetic diversity of the population (see Box 1).

### 2.2 Expression‐based approach

This approach leverages sex differences in gene expression to identify sex‐limited transcripts originating from the Y or W chromosome. RNA‐seq reads from the heterogametic sex are mapped to a reference generated from the homogametic sex. Successfully mapped reads originate from regions of the genome that are shared between the sexes whereas unmapped reads represent sex‐limited regions (Cortez et al., 2014; Moghadam, Pointer, Wright, Berlin, & Mank, 2012). These unmapped reads can be assembled de novo into potential Y‐ or W‐linked contigs. Mapping RNA‐seq reads from the homogametic sex onto these putative contigs can be used to validate sex‐limitation (Cortez et al., 2014) (Figure 2b).

This approach is similar to subtraction‐based methods employed using DNA‐seq data and is best optimized for systems with sufficiently diverged sex chromosomes or strata where there is sex‐specificity among RNA‐seq reads. Furthermore, this approach may underperform in systems where the sex chromosomes are starting to decay, as the loss of gene expression from genes on the Y or W chromosome has been shown to precede sequence degeneration (Bachtrog, 2013). Autosomal genes with sex‐limited expression may also lead to erroneous results. Therefore, while sufficient data can be obtained from as little as one male and one female, prior knowledge of when sex‐limited genes are expressed, and in which tissue, is essential to ensure detection of their associated transcripts. Typically, in heteromorphic systems, W and Y‐linked genes tend to be expressed primarily in reproductive tissue (Moghadam et al., 2012; Skaletsky et al., 2003).

### 2.3 Association‐based approach

Several approaches exist to identify sex‐linked regions using sex‐specific genetic association. While whole‐genome sequencing offers the most complete resolution for these analyses, reduced representation methods may also be employed if genotyping is sufficiently dense. Restriction site‐associated DNA sequencing (RAD‐seq) is a powerful tool to identify sex‐limited loci and has been used to infer sex chromosome systems across a number of species (Gamble et al., 2015; Jeffries et al., 2018). RAD‐seq markers are compared between males and females, and markers present in one sex and absent in the other are kept as candidate loci (Y‐specific or W‐specific; Figure 2c). Recently, this approach has been expanded to screen for variants with sex differences in allele frequency and heterozygosity (Brelsford, Lavanchy, Sermier, Rausch, & Perrin, 2017; Jeffries et al., 2018). For example, a Y‐linked allele should have a frequency of 0.5 in males versus 0 in females, and should be heterozygous in males yet homozygous in females. Therefore, this approach can be successfully applied to identify sex‐specific markers on homomorphic sex chromosomes (Gamble & Zarkower, 2014).

The inference of ploidy from RAD‐seq data can also be a fruitful avenue to identify sex‐linked regions. DetSex is a Bayesian method that infers segregation type based on ploidy information in males and females, which is derived from genotyping data (Gautier, 2014). The X chromosome is diploid in females yet haploid in males, whereas autosomes are diploid in both sexes. However, this approach assumes sex chromosomes are old and that Y reads do not map onto the X reference, and is therefore optimized for heteromorphic sex chromosomes. Furthermore, this approach requires the sequencing of many individuals (20–50 individuals). Others have leveraged RAD‐seq data to identify sex‐linked regions using GWAS, treating sex as a binary case/control variable, and using sliding window F_ST_ analysis to identify regions of genetic differentiation between males and females (Dixon, Kitano, & Kirkpatrick, 2019; Franchini et al., 2018).

The primary advantages of the RAD‐seq approach are that it relies on genomic DNA, is relatively cheap, and is highly effective for wild‐caught samples, provided they are accurately sexed. It can be used in combination with certain bioinformatic approaches to identify both homomorphic and heteromorphic sex chromosome systems, and the choice of restriction enzyme can be tailored to cut more or less frequently if the size of the nonrecombining region is known. The main challenge faced when using reduced representation methods is the problem of missing data (Lowry et al., 2017). Sex‐specific sequences are often detected in both sexes and are likely to represent false positives. A solution might be to increase sample size; however, the number of shared loci decreases with sample numbers in RAD‐seq data (Mastretta‐Yanes et al., 2015). Several studies have had success by sampling ~5–20 individuals per sex (Fowler & Buonaccorsi, 2016; Gamble et al., 2015; Gamble & Zarkower, 2014; Jeffries et al., 2018); however, false positives can also be problematic with very small numbers of males and females, and greater skew in sample sexes. Implementing and developing approaches to quantify the false positive rate of identifying sex‐linked sequences is a future priority when using this approach (see Box 1).

### 2.4 SNP density approach

While sex differences in genomic coverage or expression are indicative of diverged sex chromosomes with significant Y or W degeneration, differences in SNP density between males and females are expected in sex chromosomes at the earlier stages of divergence. In particular, elevated SNP density in the heterogametic sex can be used to infer sex‐linked regions when mapped to a reference genome generated from the homogametic sex. For example, in nascent sex chromosomes with limited Y chromosome degeneration, Y‐linked genomic reads will map to the homologous region of the X in a female reference genome, resulting in elevated SNP density in males relative to females (Figure 2d). Therefore, elevated SNP density in the heterogametic sex can be used to infer sex‐linked regions when mapped to a reference genome generated from the homogametic sex (Darolti et al., 2019; Vicoso et al., 2013; Wright et al., 2017). In contrast, in regions where the Y has largely degenerated, we expect SNP density to be lower in males when mapped to a female genome as the X is effectively hemizygous in males (Rovatsos, Farkačová, Altmanová, Johnson Pokorná, & Kratochvíl, 2019; Rovatsos, Rehák, Velenský, & Kratochvíl, 2019; Rovatsos, Vukić, & Kratochvíl, 2016). Therefore, an absence of SNPs in females can indicate X‐linked sequences. Finally, scaffolds with limited sex differences in polymorphism are probably autosomal or pseudoautosomal. Together, this rationale can be used not only to identify sex chromosomes at the intermediate stages of divergence, but also strata of different ages along the chromosome (Darolti et al., 2019; Wright et al., 2017) (Figure 2d). Contrasting SNP density between males and females is therefore a powerful approach to identify sex chromosomes or strata at the intermediate stages of X and Y (or Z and W) divergence.

The primary drawback of the SNP‐based approach is the difficulty in defining a threshold above which SNP density between males and females can be used to infer sex‐linkage. This is because the magnitude of sex differences in SNP density is directly proportional to the degree of divergence between the sex chromosomes. Therefore, implementing these approaches in young sex chromosome systems should ideally be accompanied by information as to the location of the sex determining region. Often this information is not available and therefore a permutation approach to estimate the null distribution of sex differences in SNP density across the genome is essential to identify regions with significantly elevated SNP density in the heterogametic sex (see Box 1). This method is most successful when combined with the coverage approach (Figure 2d) so that multiple, independent lines of evidence can be used to identify sex‐linked regions (Darolti et al., 2019; Shearn et al., 2019; Vicoso et al., 2013).

### 2.5 Segregation analysis approach

Segregation analyses can be a powerful approach to identify sex‐linked sequences (Bergero & Charlesworth, 2011; Chibalina & Filatov, 2011; Muyle et al., 2016). For example, SNPs in X‐linked genes will only be transmitted from the father to daughters but not sons, whereas SNPs in Y‐linked genes are only transmitted to sons. Recently, a probabilistic framework (SEX‐DETector) has been developed to infer autosomal and sex‐linked genes using patterns of allelic segregation (Muyle et al., 2016). SEX‐DETector uses genotypic data from parents and progeny to infer three segregation types: autosomal, X‐linked with a Y‐linked ortholog (X/Y pair) and those without (X‐hemizygous) (Figure 2e). Each SNP is assigned a likelihood of these three states and the method can also estimate the type of sex chromosome system through a model comparison strategy. An important step is the generation of a de novo reference assembly where X and Y sequences co‐assemble into one contig instead of separate X‐ and Y‐linked sequences. This co‐assembly makes it possible to identify X/Y SNPs and is essential for differentiating Y‐linked sequences from autosomal genes with male‐limited expression in the case of RNA‐seq data. Therefore, the approach is best optimized to systems with low or intermediate level of sex chromosome divergence where X and Y sequences are most likely to coassemble in the reference assembly. However, SEX‐DETector can still identify X‐hemizygous contigs in old systems, but there is a risk that these are actually X/Y pairs whose sequences were so diverged that they assembled into separate contigs (see Muyle et al., 2018).

This method has been used to identify sex‐linked regions in several plant species (Martin et al., 2019; Muyle et al., 2017, 2018; Veltsos et al., 2019; Zemp et al., 2016), but there are a number of important points to consider. This approach requires family data and is therefore limited to species for which pedigree information is available. Second, SEX‐Detector has primarily been used to analyse RNA‐seq derived genotyping data although it can also be used with genomic‐based data instead, providing the data set is not too big (Muyle et al., 2016). Whilst RNA‐seq data clearly has advantages, only genes that are expressed can be identified as sex‐linked. However, using multiple tissues or tissues where many genes are expressed can circumvent this problem. Finally, the pipeline requires polymorphism data to infer certain types of sex‐linkage and therefore is not optimized for inbred populations. Ideally, parents should be sampled from different populations in order to maximize the genetic diversity of the progeny and increase statistical power (but see Box 1). However, this only applies to X‐hemizygous genes, whose identification relies on the presence of polymorphisms on the X copy. The detection of X/Y gene pairs is instead based on fixed X‐Y substitutions and is therefore not affected by population levels of genetic diversity (Muyle et al., 2016, 2018). As a result, X‐hemizygous genes are sometimes more difficult to detect using this approach (Blavet et al., 2015) and this ascertainment bias should be taken into account when estimating gene loss.

BOX 1 **Overarching challenges in identifying sex chromosomes**

1 **Identifying homomorphic sex chromosomes**

Homomorphic sex chromosomes, or recently diverged strata, are challenging to identify as there is limited sequence divergence between chromosome pairs. Crucially, because homomorphic sex chromosomes can be the result of high sex chromosome turnover (Wright et al., 2016), they are precisely the systems needed to understand the mechanisms underlying the evolution of sex determination (Bachtrog et al., 2014; Beukeboom & Perrin, 2014).

A number of approaches are more suited to detecting homomorphic sex chromosomes than others. Because SNP variation accumulates before sex chromosome decay, differences in heterozygosity between males and females can be detected even when regions have not diverged sufficiently to show coverage differences (Pucholt et al., 2017). Similarly, segregation analysis approaches, such as SEX‐DETector (Muyle et al., 2016) perform optimally when X and Y chromosomes coassemble in the reference genome and are therefore best suited to detecting homomorphic sex chromosomes. Since linkage mapping directly measures recombination, this approach can also be used to identify intermediately diverged sex chromosomes; however, depending on the recombination frequency, this may have limited success in defining strata boundaries (Wright et al., 2019).

2 **Bioinformatic margins of error**

It is crucial to independently verify candidate sex‐linked regions, especially those identified using measures of sequence divergence or other proxies for arrested recombination. Although many of the methods we discuss can be implemented with small sample sizes, using fewer individuals increases the likelihood that candidate loci meet screening criteria by chance or due to sequencing artifacts. PCR amplification of candidates is a simple and widely used method of verification, however, while it is an inexpensive and straightforward method of verification, it can be prohibitively labour‐intensive for large‐scale studies. Additionally, PCR validation might fail for some loci that are surrounded by conserved sequence (Fowler & Buonaccorsi, 2016; Gamble, 2016), thus requiring additional steps toward verification.

Estimating the false positive rate using computational methods can be a complementary and alternative approach to validating sex‐linked loci. Permutation tests that shuffle sex assignments among sampled individuals are essential for generating null distributions against which to assess the validity of candidate loci (Huylmans et al., 2019; Jeffries et al., 2018; Morris et al., 2018; Scharmann, Grafe, Metali, & Widmer, 2017; Wright et al., 2017). For example, in an XY system, identifying the number of loci conforming to ZW expectations is essential to estimate the false positive rate and distinguish true sex‐linkage from stochastic noise. Alternatively, directly verifying the presence of fixed differences between males and females can be used to validate sex‐linkage of genes (Hough, Hollister, Wang, Barrett, & Wright, 2014). Bioinformatic approaches to validation such as these will be of increasing importance as data sets grow.

3 **Depth of next‐generation sequencing**

An important point to consider when designing an experiment to identify sex chromosomes is the sequencing depth. Clearly there is a trade‐off between number of individuals, which improves the likelihood of identifying sex‐linked regions particularly if the population from which they are sampled is genetically diverse, and the depth of sequencing. Deeper sequencing reduces the chances of sequencing errors leading to the misidentification of sex‐linked regions (Davey et al., 2013; Liu et al., 2012; Mastretta‐Yanes et al., 2015; Nielsen, Paul, Albrechtsen, & Song, 2011). However, the majority of approaches rely on sequencing both the homogametic sex, where the sex chromosomes will have equal depth to the autosomes, and the heterogametic sex, where the X and Y (or Z and W) chromosomes will have half the sequencing depth. For example, our recommendation of >20‐fold sequencing depth for coverage‐ and heterozygosity‐based approaches (Carvalho & Clark, 2013; Hall et al., 2013; Smeds et al., 2015; Vicoso & Bachtrog, 2015) ensures sex chromosomes are sequenced 10‐fold in the heterogametic sex.

4 **Population genetic diversity**

Approaches that rely on identifying consistent genetic differences between males and females (e.g., genomic coverage, SNP density, expression and RAD‐seq methods) to identify sex chromosomes are most accurate when inbred populations are used. This is because in outbred populations, males and females will differ by chance at polymorphic sites across the genome, making it difficult to identify sex‐linked regions, particularly when only a few individuals are sampled. In contrast, approaches that rely on patterns of SNP segregation (e.g., linkage mapping) perform optimally on outbred populations where genetic diversity is maximized. However, care must be taken if sampling across populations, as it is possible that individuals from different populations will have independently evolved sex chromosome systems which can confound the results of these approaches (discussed in Jeffries et al., 2018).

5 **Determining the identity of the sex chromosome pair**

Once sex‐linked loci are found, it is necessary to determine the identity of the sex chromosome pair in order to identify potential turnover events. This can be achieved by searching for orthologous sequences in an outgroup species with a chromosomal‐level genome assembly. This is often challenging and highly dependent on conservation of synteny across clades. However, a number of different methods are available for this purpose, including the Reference‐Assisted Chromosome Assembly (RACA) algorithm (Kim et al., 2013) as used in Darolti et al. (2019), or a custom approach developed by Jeffries et al. (2018), involving the generation of linkage maps from RAD‐seq data to anchor scaffolds to an outgroup reference genome. The importance of these algorithms, as well as the importance of generating chromosomal‐level genome assemblies in multiple species, will be a priority in order to estimate the diversity of sex chromosomes in many undersampled clades.

### 2.6 Linkage mapping approach

Instead of using a proxy for arrested recombination, such as sequence divergence or the accumulation of sex‐specific SNPs, sex chromosomes can be identified by finding regions of the genome where there is no recombination in males or females. Linkage maps measure recombination frequency between genetic makers and are a traditional method for sex chromosome discovery (Al‐Dous et al., 2011; Charlesworth, 2018; Goldberg, Spigler, & Ashman, 2010; Hou et al., 2015). The first step of this process requires DNA collection from parents and offspring. Typically, large sample sizes are required (~100s to 1,000s of progeny) from multiple independent families, where the number of individuals will determine the number of potential crossover events observed and therefore resolution to distinguish autosomal from sex‐linked regions. Therefore, when recombination is rare, even larger families are needed (Bergero, Gardner, Bader, Yong, & Charlesworth, 2019; Wright et al., 2019). Next, informative genetic markers need to be identified that are evenly spread across the whole genome, or along the sex chromosome if strata and the pseudoautosomal region are being identified (e.g., Yazdi & Ellegren, 2018). Finally, linkage maps for males and females are constructed, and regions of the genome with no recombination indicate putative sex‐linked loci (Figure 2f). Simultaneously, QTL analysis using a binary trait model could be used to quantify the number and size of the regions involved.

The advantage of linkage mapping is that it directly measures recombination rates rather than using a proxy for arrested recombination, and thus can be applied to species with homomorphic sex chromosomes. However, the necessity for samples from parents and offspring will limit which species this approach can be used on. Recombination frequency will also determine how successful this approach is. If the sex‐determining locus arose in an area of the genome which already had low recombination, as is believed to have occurred in papaya (Wai, Moore, Paull, Ming, & Yu, 2012), then sex chromosome discovery using linkage mapping will be more challenging. Furthermore, when recombination events are rare, the boundary between the nonrecombining and the pseudoautosomal regions is more difficult to define (Bergero et al., 2019; Wright et al., 2019). This is because the probability of observing a recombination event near this boundary is limited by sample size. Large families, and correspondingly many recombination events, are necessary to achieve the power required to characterize nonrecombining regions on sex chromosomes. This approach also cannot be used in species with sex‐limited recombination (e.g., several Diptera and Lepidoptera; see Satomura, Osada, & Endo, 2019 for a complete review).

## 3 FUTURE DIRECTIONS & PERSPECTIVES

The diversity of independently evolved sex chromosome systems across eukaryotes is striking (Bachtrog et al., 2014; Beukeboom & Perrin, 2014), yet our current understanding of the ecological and genetic factors that drive changes in sex determination system is limited, despite a large body of theoretical predictions. The development of new bioinformatic methods to identify and characterize sex chromosomes across non‐model species is fueling efforts to test these predictions. Indeed, several studies have recently provided important insight into the dynamics and drivers of turnover (Blackmon & Demuth, 2014; Jeffries et al., 2018; Kitano & Peichel, 2012). A large body of theoretical work outlines predictions for when and why sex chromosome transitions occur (Bachtrog et al., 2011; Beukeboom & Perrin, 2014), under the hypotheses of genetic drift (Bull & Charnov, 1977; Saunders et al., 2018), accumulation of deleterious mutation on the sex‐limited chromosomes (Blaser et al., 2013, 2014), selection on sex ratio (Jaenike, 2001; Werren & Beukeboom, 1998) and sexually antagonistic selection (van Doorn & Kirkpatrick, 2007, 2010). Here, we highlight key predictions for each of the hypotheses to motivate future sex chromosome research.

### 3.1 Genetic drift

Genetic drift has been theorized to underlie sex chromosome turnover in the absence of selection when a novel sex determining region arises of equal fitness to the established one (Bull & Charnov, 1977). The emergence of a new sex determination locus is thought to be followed by a period of multifactorial sex determination involving multiple genotypes for each sex. The two resulting sex chromosome systems are connected by a path of neutral equilibria that balance sex ratio at the population level, enabling drift to drive a transition to the new system (Bull & Charnov, 1977). Transitions that reverse patterns of heterogamety are characterized by a drift‐induced selective force that favours the fixation of novel sex determining mutations (Veller, Muralidhar, Constable, & Nowak, 2017). However, the weakness of drift‐induced selection (fixation probabilities on the order of 1/*N*) calls into question its significance in mediating turnover given the potential for other selective forces to act on competing sex chromosome systems (Veller et al., 2017). Furthermore, the coexistence of multiple sex determining loci in a number of species (e.g., cichlids, housefly, zebrafish, seabass) suggests that multifactorial sex determination need not be unstable, provided the sex ratio is balanced (Liew et al., 2012; Meisel et al., 2016; Moore & Roberts, 2013; Roberts et al., 2016; Vandeputte, Dupont‐Nivet, Chavanne, & Chatain, 2007; Wilson et al., 2014). Because sex operates as a threshold trait in which female or male development is triggered when genetic and/or environmental cues surpass some level (Bulmer & Bull, 1982; Roff, 1996), the presence of multiple sex determining loci may not necessarily indicate that a system is undergoing a sex chromosome turnover (Beukeboom & Perrin, 2014; Perrin, 2016; Rodrigues et al., 2017).

Drift‐induced turnover has been studied almost entirely using computer simulations, and this work has generated a number of predictions to guide future research (Nishioka, Miura, & Saitoh, 1993; Saunders et al., 2018; Veller et al., 2017). First, drift‐induced sex chromosome transitions that maintain patterns of heterogamety are predicted to be 2–4 times more likely than those which reverse heterogamety when the invading sex determining locus is dominant; however, this ratio is influenced by effective population size and mating system. This is because transitions that preserve heterogamety involve fixation of the ancestral X or Z chromosome, which have a higher frequency in the population, while transitions reversing heterogamety require fixation of the ancestral Y or W (Saunders et al., 2018). Comparative studies across independently evolved sex chromosomes offer the potential to test this directly, provided that the sampling resolution is sufficient and the identity of sex chromosome pairs is known. The preserved heterogamety patterns among Salmonid fish (Phillips, 2013), Varanid and Lacertid lizards (Ezaz, Sarre, O'Meally, Graves, & Georges, 2009; Pokorná & Kratochvíl, 2009), and Ranid frogs (Jeffries et al., 2018) are consistent with drift‐induced turnover, but are difficult to distinguish from expectations under alternative scenarios such as mutation‐load selection (Jeffries et al., 2018). However, the predictions of mutation‐load models rely on explicitly accounting for mutation rates, which can be challenging to obtain. Second, while transitions that maintain heterogamety are unaffected by demographic parameters, transitions that reverse heterogamety are more likely as effective population size decreases and reproductive skew increases (Saunders et al., 2018; Veller et al., 2017). Specifically, transitions from an XY to a ZW system become more common when the number of breeding males is low (Saunders et al., 2018). Therefore, experimental and comparative approaches in species with multifactorial systems may present a window into an ongoing turnover event, and offer an excellent opportunity to explicitly test the role of drift in sex chromosome turnover. Under drift, multifactorial systems should be found more frequently in species with large effective population sizes because the fixation of an invading sex determiner will proceed more slowly in such species (Saunders et al., 2018; Veller et al., 2017). Natural or experimentally induced variation in demographic traits and mating systems, and thereby effective population size, across species can be used to probe the role of drift in driving turnovers. Finally, directly identifying invading sex determiners makes it possible to test the prediction that heterogamety‐reversing transitions should involve dominant mutations (Nishioka et al., 1993; Veller et al., 2017).

### 3.2 Accumulation of deleterious mutations

As recombination is suppressed between sex chromosomes, the sex‐limited Y and W start to decay by a combination of neutral and adaptive processes. The accumulation of loss‐of‐function mutations on the nonrecombining sex chromosomes is predicted to drive the turnover and formation of a new sex chromosome system. This process is thought to be affected by the number and strength of deleterious mutations, sexually antagonistic selection, effective population size, and the size of the nonrecombining region (Blaser et al., 2013, 2014).

A number of predictions for sex chromosome turnover arise from the mutation accumulation hypothesis. First, patterns of heterogamety should be preserved, because a switch (e.g., from an XY to a ZW system) requires the fixation of the ancestral, degenerated sex‐limited chromosome as an autosome (Blaser et al., 2014; van Doorn & Kirkpatrick, 2010; Jeffries et al., 2018; Scott, Osmond, & Otto, 2018). Second, factors associated with high loads of deleterious mutations, and therefore sex chromosome degeneration, should also be linked to high turnover rates. Many species exhibit heterochiasmy or achiasmy, where recombination is reduced or absent in one sex, which would in theory accelerate the accumulation of deleterious mutations on the nonrecombining sex chromosome and therefore promote turnover. This is consistent with transitions across Ranid frogs (Jeffries et al., 2018) but not with the stability of ZW chromosomes in Lepidoptera (Lenormand, 2003), both of which exhibit reduced or absent recombination in the heterogametic sex. Various life history traits can also be used as a proxy of mutation rate and therefore sex chromosome degeneration in a comparative framework. For example, species that are warm blooded, shorter lived, or have a smaller body size usually have higher metabolic rates (Galtier, Jobson, Nabholz, Glémin, & Blier, 2009). However, current studies find that many cold‐blooded vertebrates including fish (Mank & Avise, 2009; Mank et al., 2006; Volff, Nanda, Schmid, & Schartl, 2007), reptile, and amphibian lineages (Ezaz et al., 2009; Jeffries et al., 2018) have undergone far more sex chromosome turnover than warm‐blooded mammals. This contrast may reflect the confounding effects of other factors, such as differences in effective population size. In addition, organisms with a longer haploid phase will experience purifying selection to maintain gene activity on the Y chromosome during meiosis (Wright et al., 2016). Therefore, we might expect less frequent sex chromosome turnover in organisms where haploid selection is more persistent. However, whilst it was initially shown that organisms with a long haploid phase exhibit lower levels of sex chromosome divergence, including some algae (Ahmed et al., 2014) and plants (Bergero, Qiu, & Charlesworth, 2015; Chibalina & Filatov, 2011), a recent study using a larger data set of sex‐linked genes found rapid degeneration of the *Silene latifolia* Y chromosome (Papadopulos, Chester, Ridout, & Filatov, 2015). This result, together with the observation that many plant clades exhibit turnover of sex chromosome systems (Balounova et al., 2019; Charlesworth, 2015; Martin et al., 2019; Moore, Harkess, & Weingartner, 2016; Tennessen et al., 2018), suggest that haploid selection might have a minimal effect on rates of Y degeneration.

Finally, the rate of turnover of XY versus ZW chromosomes is predicted to differ in light of mutation load. First, the evolution of complete dosage compensation, a mechanism that compensates for the degeneration and loss of expression of the W and Y chromosomes (Gu & Walters, 2017; Mank, 2013), is thought to reduce the power of purifying selection to maintain gene activity on these chromosomes (Engelstädter, 2008; Wright et al., 2016). Dosage compensation mechanisms are more frequently observed on XY relative to ZW chromosomes in the species studied so far (Gu & Walters, 2017; Mullon et al., 2015; Tables S1–S3), potentially leading to faster rates of Y chromosome decay. However, there have been several recent counter‐examples to this trend (Hale, McKinney, Thrower, & Nichols, 2018; Huylmans et al., 2019), and as more sex chromosomes are identified it will be possible to test whether there is indeed a consistent relationship between dosage compensation status and sex chromosome system. Second, in several vertebrate and plant groups (Kirkpatrick & Hall, 2004; Whittle & Johnston, 2002), males have a higher mutation rate than females. Therefore, deleterious mutations are predicted to accumulate more quickly on the Y chromosome, meaning that XY sex chromosome systems may undergo turnover more often than ZW systems (Bachtrog et al., 2011; Naurin, Hansson, Bensch, & Hasselquist, 2010). Testing this directly will require detailed knowledge of the identity of the sex chromosome pair across multiple species.

### 3.3 Selection on sex ratio

Selection on sex ratio is thought to promote the invasion of a novel sex determination locus in order to restore Fisherian sex ratio values when they are unbalanced (Beukeboom & Perrin, 2014; Bull, 1983; Mank, Hosken, & Wedell, 2014). This can arise commonly through intragenomic conflicts from selfish or meiotic drive elements, either autosomal or sex‐linked. Endosymbionts can have a similar impact, as illustrated by the *Wolbachia* feminizing element in populations of woodlice (Cordaux, Bouchon, & Grève, 2011). Increasing numbers of theoretical models outline the scenarios in which we might expect sex ratio selection to drive the evolution of new sex chromosome systems (Kozielska, Weissing, Beukeboom, & Pen, 2010; Úbeda, Patten, & Wild, 2015) and there is growing support from a few taxa (Badawi, Moumen, Giraud, Grève, & Cordaux, 2018; Becking et al., 2017; Chebbi et al., 2019; Cordaux et al., 2011; Cordaux & Gilbert, 2017; Leclercq et al., 2016; Miura, 2007). Similarly, a recent study outlined the role of haploid selection via gametic competition and meiotic drive in increasing the lability of sex determination systems (Scott et al., 2018).

Given the prevalence of sex ratio distorters in nature (Hall, 2004; Jaenike, 2001), in particular sex‐linked meiotic drivers (Helleu et al., 2016; Tao et al., 2007), sex ratio selection is likely to be a common driver in sex chromosome turnover events (see Scott et al., 2018), yet is probably one of the most difficult to detect due to its transient nature (Kozielska et al., 2010). This is because once the novel sex determination region is fixed, balanced sex ratios are restored and the original sex determining locus is often lost from the population. As a result, comparative phylogenetic approaches will have limited power to quantify the relative contribution of meiotic drive to turnover events. However, one signature of a recurrent arms race between successive sex ratio distorters and their modifiers is an increase in the length of the sex determination pathway, as novel sex determination factors are integrated into existing gene networks (Schartl, 2004; Wilkins, 1995). In support of this, downstream components of sex determination cascades are broadly conserved relative to upstream regulators (Beukeboom & Perrin, 2014). Alternatively, laboratory crosses between pairs of sister species can uncover the potential for sex ratio selection to act by uncoupling drivers and modifiers; however, such experiments are not feasible in many groups. Instead, experimental selection in species with polyfactorial sex determination, such as the housefly (Kozielska, Pen, Beukeboom, & Weissing, 2006; Meisel, Olafson, Guerrero, Konganti, & Benoit, 2019), have the greatest scope to quantify the role of sex ratio selection and meiotic drive in the evolution of sex determination.

### 3.4 Sexually antagonistic selection

Sexually antagonistic selection, which occurs when a mutation is harmful to one sex but beneficial to the other, is predicted to drive sex chromosome turnover. For example, an autosomal gene with male benefit and female harm effects might become linked to a sex determining gene, either through the evolution of a novel locus or translocation of the existing determiner or antagonistic locus. If this neo‐sex chromosome produces males with higher fitness than the ancestral Y chromosome, then it can replace the ancestral sex determination mechanism (van Doorn & Kirkpatrick, 2007, 2010).

There is some empirical support for this theory, including the invasion of a novel female sex determining locus in cichlids where there is sexual conflict over a female‐benefit, male‐harming colour pattern (Roberts, Ser, & Kocher, 2009). However, since we can only look at a snapshot in evolutionary time, and given that sex determination is dynamic and polygenic in cichlids (Ser, Roberts, & Kocher, 2010), we do not know whether the new sex chromosome predates, or evolved in response to, the coloration patterns. The discovery of a neo‐sex chromosome in the three‐spined stickleback also supports models of sex chromosome evolution driven by sexual antagonism (Kitano et al., 2009), however, the absence of recombination suppression between the sexually antagonistic locus and the sex determining gene casts doubt on this (Natri, Shikano, & Merilä, 2013). Finally, sexually antagonistic genes have accumulated close to a novel sex determining gene (Rice, 1992) and on a neo‐sex chromosome in *Drosophila* (Zhou & Bachtrog, 2012). Despite these studies, we lack direct support for the relative importance of sexual antagonism in driving turnovers. One way around this is through experimental evolution, and an ambitious study, involving 100 generations of backcrossing between two species of *Xiphophorus*, directly illustrates the potential for sexual conflict to drive sex chromosome turnover (Franchini et al., 2018).

Much of the current work in this area involves species of fish, and we suggest future work should continue in these taxa due to the repeated origins of homomorphic sex chromosomes. Studying species or populations where there is variation in the extent of recombination suppression between sex chromosomes, as in Poeciliids (Bergero et al., 2019; Darolti et al., 2019; Wright et al., 2017), promises to be a fruitful avenue. A powerful approach would be targeting young sex chromosomes within a sex‐specific evolution framework to test whether sexually antagonistic mutations accumulate prior to recombination suppression (Ponnikas, Sigeman, Abbott, & Hansson, 2018). Experimental evolution continuing the work of Rice (1992), investigating whether recombination suppression spreads between a new sex determining gene and a sexually antagonistic gene would be an insightful, although challenging, future avenue.

## AUTHOR CONTRIBUTIONS

All authors were responsible for writing the manuscript.

## Data Availability

Data sharing is not applicable to this article as no new data were created or analyzed in this study.
